# Disseminating information on acute public health events globally: experiences from the WHO’s Disease Outbreak News

**DOI:** 10.1136/bmjgh-2023-012876

**Published:** 2024-02-26

**Authors:** Harsh Lata, Neil Jan Saad Duque, Eri Togami, Alessandro Miglietta, Devin Perkins, Aura Corpuz, Masaya Kato, Amarnath Babu, Tshewang Dorji, Tamano Matsui, Maria Almiron, Ka Yeung Cheng, Lauren E MacDonald, Jukka Tapani Pukkila, George Sie Williams, Roberta Andraghetti, Carmen Dolea, Abdirahman Mahamud, Oliver Morgan, Babatunde Olowokure, Ibrahima Socé Fall, Adedoyin Awofisayo-Okuyelu, Esther Hamblion

**Affiliations:** 1 Health Emergencies, World Health Organization, Geneva, Switzerland; 2 Health Emergencies, World Health Organization Regional Office for the Eastern Mediterranean, Cairo, Egypt; 3 World Health Organization Regional Office for South-East Asia, New Delhi, Delhi, India; 4 Health Emergencies, World Health Organization Regional Office for South-East Asia, New Delhi, Delhi, India; 5 World Health Organization Regional Office for the Western Pacific, Manila, The Philippines; 6 Health Emergencies, Pan American Health Organization, Washington, District of Columbia, USA; 7 Health Emergencies, World Health Organization Regional Office for Europe, Copenhagen, Denmark; 8 Health Emergencies, World Health Organization Regional Office for Africa, Brazzaville, Congo; 9 Health Emergencies, World Health Organization Regional Office for the Western Pacific, Manila, The Philippines

**Keywords:** Epidemiology, Public Health, Infections, diseases, disorders, injuries

## Abstract

WHO works, on a daily basis, with countries globally to detect, prepare for and respond to acute public health events. A vital component of a health response is the dissemination of accurate, reliable and authoritative information. The Disease Outbreak News (DON) reports are a key mechanism through which WHO communicates on acute public health events to the public. The decision to produce a DON report is taken on a case-by-case basis after evaluating key criteria, and the subsequent process of producing a DON report is highly standardised to ensure the robustness of information. DON reports have been published since 1996, and up to 2022 over 3000 reports have been published. Between 2018 and 2022, the most frequently published DON reports relate to Ebola virus disease, Middle East respiratory syndrome, yellow fever, polio and cholera. The DON web page is highly visited with a readership of over 2.6 million visits per year, on average. The DON report structure has evolved over time, from a single paragraph in 1996 to a detailed report with seven sections currently. WHO regularly reviews the DON report process and structure for improvements. In the last 25 years, DON reports have played a unique role in rapidly disseminating information on acute public health events to health actors and the public globally. They have become a key information source for the global public health response to the benefit of individuals and communities.

**SUMMARY BOX**

- Dissemination of information on acute public health events is an essential feature of a response.
- WHO communicates through a variety of means, including the Disease Outbreak News (DON) report.
- This article provides an overview of DON reports published between 1996 and 2022, elucidates the criteria and the decision-making process, and outlines improvements to the DON report over time.
- An improved understanding of acute public health events for which DON reports are published and clarification of the process will improve comprehensiveness, enhance transparency and further trust in an age where misinformation and disinformation for acute public health events are widespread.

## Introduction

Information dissemination is a critical part of public health intelligence. Disseminating accurate and timely information on acute public health events is an essential part of response measures.^1–5^ Rapid, clear and substantive information sharing on the occurrence, timing, location and affected population, as well as recommended actions, guides decision-making, builds trust between the public and health actors, and can save lives.^6 7^

WHO has a constitutional mandate to disseminate authoritative and independent information on acute public health events that may constitute a public health emergency of international concern (PHEIC).^8^ This mandate is strengthened by the International Health Regulations 2005 (IHR 2005), a legally binding agreement between WHO and 196 States Parties across the world, including all 194 WHO Member States.^9^ Under IHR (2005), States Parties agree to strengthen surveillance and response activities; it also requires WHO to share information with both States Parties and the public on acute public health threats (box 1).

Box 1 **International Health Regulations (2005)**

- The International Health Regulations (IHR 2005) is a legally binding agreement for 196 countries, including all 194 Member States of WHO. The aim of IHR (2005) is to provide a global framework for the international community to prevent, prepare for and respond to acute public health risks.^9^
- There are several articles which pertain to the detection, verification and information sharing of acute public health events. For information sharing, WHO has the mandate to communicate with countries (referred to as States Parties) and the public under Article 11 ‘Provision of information by WHO’ of the IHR.
- WHO communicates with countries through publications on the Event Information Site platform in accordance with Article 11.1: ‘WHO shall send to all States Parties and, as appropriate, to relevant intergovernmental organizations, as soon as possible and by the most efficient means available, in confidence, such public health information which it has received under Articles 5 to 10 inclusive which is necessary to enable States Parties to respond to a public health risk. WHO should communicate information to other States Parties that might help them in preventing the occurrence of similar incidents’.
- The criteria for the sharing of information is governed by Article 11.2: ‘WHO shall use information received under Articles 6 and 8 and paragraph 2 of Article 9 for verification, assessment and assistance purposes under these Regulations and, unless otherwise agreed with the States Parties referred to in those provisions, shall not make this information generally available to other States Parties, until such time as:
  - the event is determined to constitute a public health emergency of international concern in accordance with Article 12; or
  - information evidencing the international spread of the infection or contamination has been confirmed by WHO in accordance with established epidemiological principles; or
  - there is evidence that:
    - control measures against the international spread are unlikely to succeed because of the nature of the contamination, disease agent, vector or reservoir; or
    - the State Party lacks sufficient operational capacity to carry out necessary measures to prevent further spread of disease; or
  - the nature and scope of the international movement of travellers, baggage, cargo, containers, conveyances, goods or postal parcels that may be affected by the infection or contamination requires the immediate application of international control measures’.
- WHO communicates with the public through DON reports in accordance with Article 11.4: ‘When information received by WHO under paragraph 2 of this Article is made available to States Parties in accordance with these Regulations, WHO may also make it available to the public if other information about the same event has already become publicly available and there is a need for the dissemination of authoritative and independent information’.

In order for WHO to meet its IHR obligations and to facilitate information sharing, WHO has established an IHR Event Information Site (EIS) with restricted access. This site is accessible to all the National IHR Focal Points (defined in IHR (2005) as ‘the national centre, designated by each State Party, which shall be accessible at all times for communication with WHO IHR Contact Points…’) and provides timely information on ongoing public health events of international concern. Similarly, to provide information to the public, IHR (2005) allows WHO to share information through Disease Outbreak News (DON) reports. DON reports have been published since 1996 and are published on a dedicated WHO web page.^10^ They provide verified information on confirmed or potential acute public health events that have significant public health implications or the potential to become a PHEIC. While DON reports are available to the public, the primary audiences are health professionals, subject matter experts and the media. In addition to EIS postings and DON reports, WHO communicates regularly with WHO Member States, as well as with partners, media and the public, through (situation) reports, dashboards, press conferences and social media.^11–26^

Here, we provide an overview of DON reports published between 1996 and 2022. We also outline the decision-making criteria, elucidate the process for producing a DON report and provide insight on the changes to the DON reports over time.

## Overview of published Disease Outbreak News reports

**Figure 1:**
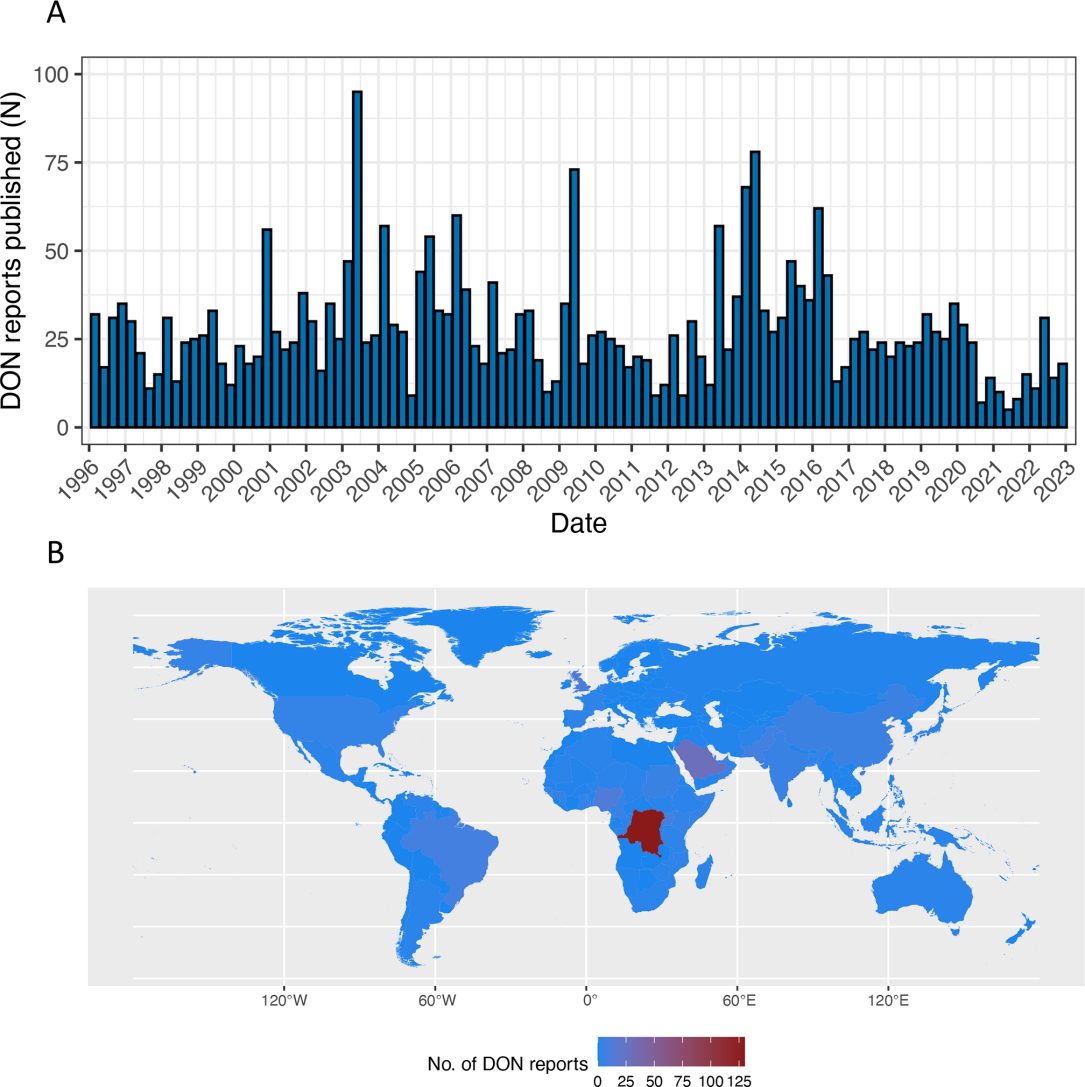
Disease Outbreak News (DON) reports: (A) number of reports published by quarter, 1996–2022, and (B) number of reports published by geographical area, 2018–2022.

DON reports have been published consistently for over 25 years and have served as one of the primary means for WHO to communicate with the public. Between 1996 and 2022, 3022 DON reports were published, which ranged from 38 in 2021 to 206 in 2014, with a median of 111 reports annually. During substantive and protracted public health events, such as the 2013–2016 Ebola virus disease outbreak in West Africa and the 2015–2016 Zika virus disease outbreak, the annual number of DON reports increased because frequent DON reports were published on these events to keep the public abreast of the latest information (figure 1A).

In 2018–2022, DON reports were published on events in 87 different countries, territories, areas or regions. In this period, the five most frequent events for which DON reports were published were Ebola virus disease (n=127, 32%), Middle East respiratory syndrome (MERS) (n=45, 11%), polio (n=29, 7%), yellow fever (n=29, 7%) and cholera (n=22, 6%). The Democratic Republic of the Congo (n=130, 33%), Saudi Arabia (n=29, 7%), Nigeria (n=13, 3%), the UK (n=10, 3%) and Brazil (n=8, 2%) were the five countries that accounted for the highest number of DON reports between 2018 and 2022 (figure 1B).

The DON report web page is one of the most read pages on WHO website, with on average more than 2.6 million visits per year between 2018 and 2022. Moreover, DON reports are translated and made available in all six United Nations (UN) languages, including Arabic, Chinese, English, French, Russian and Spanish, to improve accessibility of information. The DON report website is also linked to a public WHO Health Emergency Dashboard, which provides a global overview of acute public health events.^27^

For health events which constitute a PHEIC, or those that require regular updates amidst a rapidly evolving epidemiological situation, frequent DON updates are published as new information becomes available. For certain events such as the COVID-19 pandemic and the 2022 multicountry outbreak of mpox, the initial DON reports transitioned to specific epidemiological or operational reports from 21 January 2020 and 6 July 2022, respectively.^28 29^

## Decision-making criteria

WHO makes a decision to publish a DON report using a set of criteria. Under IHR (2005), States Parties are obliged to notify WHO on acute public health events using the decision instrument in Annex 2 if at least two of the four questions in this annex are answered ‘Yes’. These four questions include (1) whether the event has a serious public health impact, (2) is unusual or unexpected, (3) has a significant risk of international spread and (4) whether there is a risk of travel or trade restrictions. However, any of the four conditions, including (1) smallpox, (2) poliomyelitis due to wild type, (3) human influenza caused by a new subtype and (4) severe acute respiratory syndrome, are always notifiable under IHR (2005) in all circumstances.

**Figure 2:**
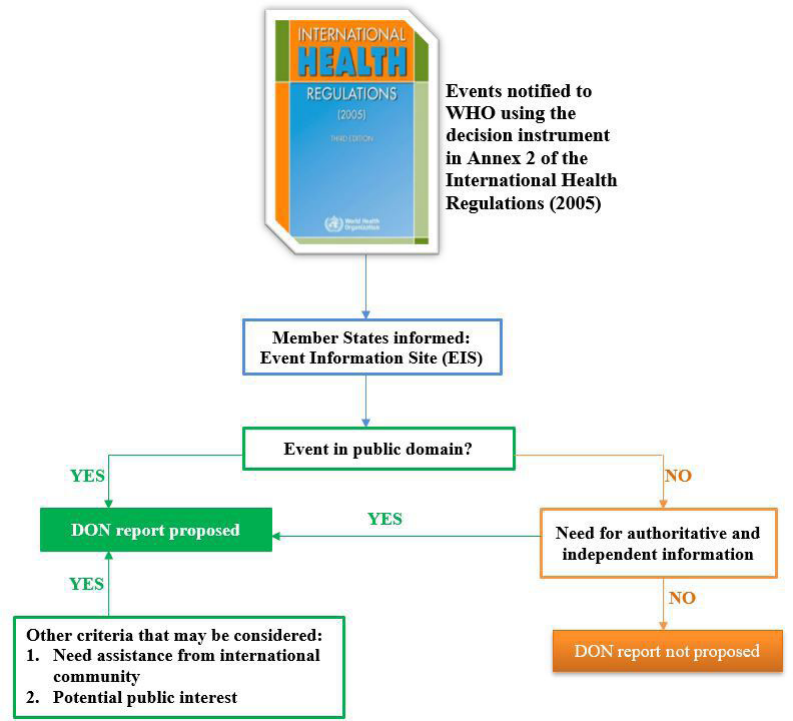
Decision instrument for producing Disease Outbreak News (DON) reports.

Once a decision is made to publish an EIS posting, several additional criteria are considered for a DON report to be published (figure 2). These include whether there is a need for assistance from the international community, potential public interest, whether information on the event has already been reported publicly and the need to disseminate authoritative and independent information as per Article 11 of IHR (2005).

Generally, a DON report is envisaged when some of the criteria are met and the information has already been made available to States Parties through an EIS posting. The States Parties affected by the event may also be informed that a DON report is in process and will be publicly disseminated via the WHO website. For prolonged ongoing acute public health events, for example the Ebola virus disease outbreak in the Democratic Republic of the Congo in 2018–2020 or MERS in Saudi Arabia, regular DON updates were produced, with or without regular EIS postings.

## Process of producing a Disease Outbreak News report

**Figure 3:**
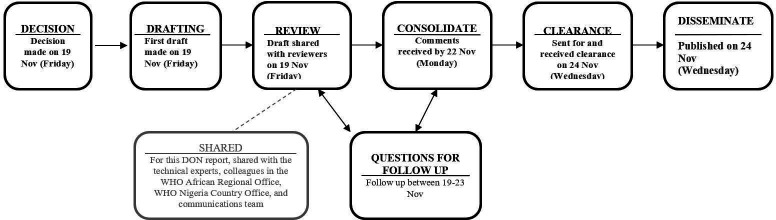
Disease Outbreak News (DON) process illustrated with timeline for a yellow fever outbreak in Nigeria in November 2020.^40^

There is a standard operating procedure for producing DON reports, comprising six stages (an example is illustrated in figure 3). Once the decision is taken to produce a DON report, a draft, generally developed drawing on the information in the EIS, is produced by a team of epidemiologists at WHO. Not all information from the EIS posting that has been shared by WHO Member States under the IHR mechanism will necessarily feature in the DON report. The draft includes technical information (see Structure of a Disease Outbreak News report section) based on discussions with subject matter experts at WHO and relevant information from situation reports or other information sources, such as official sources from the Ministry of Health.

The draft is then shared with epidemiologists, public health specialists, subject matter experts and communications experts within WHO for feedback and editing with a deadline. In the fourth step, the feedback is reviewed to ensure that the content is factually correct, up-to-date and consolidated. After incorporating the comments and feedback, a revised version is shared with those who provided prior input for final review and addressing any pending clarifications. This consolidated version is then sent for clearance by a senior epidemiologist. The DON report is disseminated by publishing on the WHO website after clearance as the final step. The DON report is also circulated by email, including to all WHO Members States as well as various partners, and added to the WHO Health Emergency Dashboard for public access.^27^ Moreover, DON reports are translated and published in all six official UN languages. In case of any factual errors discovered after the publication, WHO updates the published version and issues an erratum for the DON report.

If it has been determined that a DON report is required, WHO aims to publish it in a timely manner. For certain public health events that contain sensitive information, or if the details of the event are challenging to verify, it might require more time to publish a DON report to make sure that the information is accurate. Nevertheless, WHO also communicates urgent information to the wider public health community (eg, through a Global Outbreak Alert and Response Network (GOARN) Weekly Operations call and the secure GOARN Knowledge Platform) and the public through other means (including social media postings, press releases or press conferences) while a DON report is still in process of publication.

## Structure of a Disease Outbreak News report

The first DON report, published on 22 January 1996, consisted of a single paragraph which described an outbreak of cholera in Burundi.^30^ The structure of DON reports has changed over time to better reflect and communicate information for acute public health events. These include adding sections on the public health response (by the respective national authorities, in support and coordination with WHO and partners) and recommended public health advice from WHO. Gradually, more details of the event were added to make the DON reports more informative.

The structure and process of DON reports are regularly reviewed. The last review was conducted in 2019 prior to the COVID-19 pandemic in which 68 people (including external public health professionals, medical professionals, academic researchers and news journalists) participated. The review identified the inclusion of maps and graphs, as well as having a short ‘at a glance’ summary for each DON report to improve the readability and richness of the information. Based on these recommendations, additional sections have been included from 2022 onwards. Currently, a DON report consists of seven sections, namely situation at a glance, description of the situation, epidemiology of the disease, public health response, WHO risk assessment, WHO advice and further information (table 1).

**Table 1:** Outline and description of current Disease Outbreak News structure

Section	Description
Situation at a glance	Short summary of the situation reported in the Disease Outbreak News reports, allowing readers to immediately grasp key messages.
Description of the situation	Details about the situation with detailed epidemiological data and epidemiological curve, graphs and maps.
Epidemiology of the disease	For acute public health events due to infectious diseases. The section includes information on causative agent, mode of transmission, incubation period, symptoms and treatment.
Public health response	Outlines the (planned or implemented) actions in response to the situation by the concerned authorities and the support, or planned support, provided by WHO and/or partners.
WHO risk assessment	Summary of WHO’s independent risk assessment. Focuses on the potential risk for human health and the risk of the event spreading further, considering the context and relevant response capacities.
WHO advice	Details the public health advice in relation to the situation, as well as country and event-specific travel advice, if applicable.
Further information	Provides references and/or web links to other relevant information such as factsheets, technical guidelines and/or websites of key partners with information on the event.

## Challenges and future opportunities

The DON reports were first published in the early days of the digital age in the late 1990s and have since evolved into a key feature for dissemination of information on acute public health events globally. At the same time, the rapid global expansion of the internet and the emergence of digital and social media have transformed the way in which society interacts with information. Information on acute public health events is more available and widely disseminated than ever before, but such information is not always verified or accurate. Furthermore, disinformation and misinformation are actively occurring, including during and in response to public health events.^31–33^ As an important counterbalance, DON reports aim to provide accurate, timely and authoritative information.

The need to ensure DON reports have the most accurate information possible is underpinned by a meticulous process for confirming event details. It is necessary to involve responders who are managing and supporting the response to a public health event. Gathering information from those involved in the response, including national authorities and WHO, improves the information shared but can result in delays in publishing. WHO continuously strives to balance the need for timely publication with a requirement to publish verified public health information. However, WHO reviews the timeliness of DON reports monthly to identify delayed publishing of DON reports, putative causes as well as ways to mitigate such delays.

The structure and format of the DON reports have evolved over time, with new types or modes of information to be reported for public health events’ response. One highlight is the increased use of visualisations, such as epidemiological curves, for better illustration of the latest epidemiological trend. However, due to differential and incompatible information received, or information that includes the risk of personal identification (eg, in an outbreak with very few cases), it is not always possible to include such information in the reports. In addition, timely dissemination of DON reports takes precedence over the inclusion of new visualisations, in particular if these can delay the dissemination due to limited or non-timely availability of demographic or epidemiological information.

DON reports also serve as a publicly available repository of global acute public health events, with each of the DON reports containing detailed information about the event. This has been leveraged by health professionals and researchers as a resource on public health events.^34–38^ Therefore, to facilitate this, WHO has recently added a search function to the WHO DON web page to enable the search of DON reports by place, time and disease/events through detailed indexing.

Finally, to enhance dissemination efforts, a summary of published DON reports is featured in other WHO publications, such as the Weekly Epidemiological Record^39^ and the WHO annual reports, which have a different target audience. This can further both the number and the geographical diversity of readers that receive information on acute public health events, ensuring information of new and ongoing public health events, particularly advice on prevention and treatment, is widely disseminated.

## Conclusion

DON reports are key to sharing information on acute public health events with the public. WHO will continue to improve the DON report and strive to provide information to those in need or involved in health action. Accurate, timely and robust information sharing from DON reports enables countries, authorities and individuals to take preventive actions or to respond rapidly to acute public health events—a true global public good for health.

## Data Availability

Data are available in a public, open access repository.
